# Microbial Profiling of Buffalo Mozzarella Whey and Ricotta Exhausted Whey: Insights into Potential Probiotic Subdominant Strains

**DOI:** 10.3390/microorganisms13081804

**Published:** 2025-08-01

**Authors:** Andrea Bonfanti, Romano Silvestri, Ettore Novellino, Gian Carlo Tenore, Elisabetta Schiano, Fortuna Iannuzzo, Massimo Reverberi, Luigi Faino, Marzia Beccaccioli, Francesca Sivori, Carlo Giuseppe Rizzello, Cristina Mazzoni

**Affiliations:** 1 Department of Biology and Biotechnology “C. Darwin”, Sapienza University of Rome, 00185 Rome, Italy; andrea.bonfanti@uniroma1.it; 2 Laboratory Affiliated with the Institute Pasteur Italy and Cenci Bolognetti Foundation, Department of Drug Chemistry and Technologies, Sapienza University of Rome, 00185 Rome, Italy; romano.silvestri@uniroma1.it; 3 NGN Healthcare-New Generation Nutraceuticals s.r.l, Torrette via Nazionale 207, 83013 Mercogliano, Italy; ettore.novellino@unicatt.it (E.N.); elisabettaschiano@gmail.com (E.S.); 4 Department of Medicine and Surgery, Catholic University of the Sacred Heart, 00168 Rome, Italy; 5 Department of Pharmacy, University of Naples Federico II, 80131 Naples, Italy; giancarlo.tenore@unina.it; 6 Department of Pharmacy, University of Chieti-Pescara G. D’Annunzio, 66100 Chieti, Italy; fortuna.iannuzzo@unich.it; 7 Department of Environmental Biology, Sapienza University of Rome, 00185 Rome, Italy; massimo.reverberi@uniroma1.it (M.R.); luigi.faino@uniroma1.it (L.F.); marzia.beccaccioli@uniroma1.it (M.B.); 8 Microbiology and Virology Unit, San Gallicano Dermatological Institute, IRCCS (Istituti di Ricovero e Cura a Carattere Scientifico), 00144 Rome, Italy; francesca.sivori@ifo.it

**Keywords:** probiotics, subdominant species, metagenome, waste, food sustainability

## Abstract

Buffalo mozzarella cheese whey (CW) and ricotta cheese exhausted whey (RCEW) are valuable by-products of the Mozzarella di Bufala Campana PDO production chain. This study characterized their microbial communities using an integrated culture-dependent and -independent approach. Metabarcoding analysis revealed that the dominance of lactic acid bacteria (LAB), including *Streptococcus thermophilus, Lactobacillus delbrueckii,* and *Lactobacillus helveticus*, alongside diverse heat-resistant yeasts such as *Cyberlindnera jadinii*. Culture-based isolation identified subdominant lactic acid bacteria strains, not detected by sequencing, belonging to *Leuconostoc mesenteroides*, *Enterococcus faecalis*, and *Enterococcus durans*. These strains were further assessed for their probiotic potential. *E. faecalis* CW1 and *E. durans* RCEW2 showed tolerance to acidic pH, bile salts, and lysozyme, as well as a strong biofilm-forming capacity and antimicrobial activity against *Bacillus cereus* and *Staphylococcus aureus*. Moreover, bile salt resistance suggests potential functionality in cholesterol metabolism. These findings support the potential use of CW and RCEW as reservoirs of novel, autochthonous probiotic strains and underscore the value of regional dairy by-products in food biotechnology and gut health applications.

## 1. Introduction

Buffalo (*Bubalus bubalis*) milk, characterized by a higher content of proteins, fats, and vitamins A and D compared with cow’s milk [1], is widely used in the production of buffalo mozzarella cheese, whose market is growing internationally [2] due to its unique flavor, creamy texture, and culinary popularity. The annual production of buffalo milk in Southern Italy, currently the most important production area in the world, is approximately 200,000 tons (CLAL DATA) [3]. In such areas, the production of Campania buffalo mozzarella cheese, with a protected designation of origin (PDO), is carried out according to a specific regulation (G.U. No. 258, 6-11-2003), established to ensure the authenticity and distinctive quality of the product. The regulation designates the PDO “buffalo mozzarella” as “Campana” due to historical reasons linked to its main traditional production areas (Caserta and Salerno, as well as parts of the provinces of Naples and Benevento, all located in the Campania region). However, it extends the currently authorized production area to include parts of the provinces of Latina, Frosinone, and Rome (in the Lazio region), as well as certain areas in Puglia and Molise (specifically the province of Foggia and the municipality of Venafro). The buffalo milk is coagulated using natural calf rennet, and the curd is then broken into small grains and left to ferment for 4–5 h at 35–37 °C (curd ripening). The fermented curd is cut, melted, and stretched using hot water (90 °C). To promote milk fermentation before coagulation, the use of natural whey starter (“*siero innesto”*, locally known as “*cizza*”) is mandatory in the production of Mozzarella di Bufala Campana DOP, as it is responsible for the unique flavor and texture of the cheese. The natural whey starter is obtained through the spontaneous fermentation (6–12 h) of the whey, which is separated from the curd before melting, according to the production specification of the protected designation of origin (PDO) “Mozzarella di Bufala Campana” [4].

A significant portion of mozzarella cheese whey (CW), considered a secondary raw material, is used to produce ricotta cheese through the thermally derived flocculation (90 °C) of the whey proteins that are separated from the aqueous part, the ricotta cheese exhausted whey (RCEW). RCEW contains a considerable amount of lactose and is typically considered a final waste by-product from the whole production process.

Overall, both CW and RCEW harbor complex microbial communities composed of bacteria and yeasts originating from the native milk microbiota and environmental contamination, whose species balance is strongly influenced by the conditions of dairy processing [5]. In the specific case of the microbiota associated with buffalo milk, several bacterial and fungal strains have already been identified as having potential probiotic activity [6].

The dominant lactic acid bacteria species characterizing the buffalo natural whey starter, CW, and RCEW are *Streptococcus thermophilus*, *Lactobacillus delbrueckii*, and *Lactobacillus helveticus* [7]. While the functional and technological potentials of dominant LAB species have been extensively investigated [8], subdominant bacterial populations remain poorly studied, particularly with regard to their potential biotechnological and probiotic applications.

The subdominant microbial populations of fermented foods and beverages, correspond to the microorganisms that are present in lower numbers compared with the dominant species. In foods produced using natural starters, which are characterized by a very high biodiversity, subdominant microbiota remain partially underexplored [9,10] due to the limitations of both culture-dependent and culture-independent approaches. These include challenges in isolation and cultivation (in the former) as well as limitations such as sequencing depth and primer bias (in the latter). Although present in low abundance, it was hypothesized that subdominant microorganisms could play crucial roles in food fermentation processes by contributing to flavor and aroma development, producing bioactive or antimicrobial compounds, enhancing microbial stability, and interacting synergistically with dominant species [11]. Accordingly, subdominant strains may possess probiotic properties [12] or become functionally relevant under specific environmental conditions, making them valuable yet underexploited resources in food biotechnology [13].

In this study, the microbiota of cheese whey (CW) and ricotta cheese exhausted whey (RCEW), derived from Campania buffalo mozzarella cheese production, was investigated using an integrated culture-dependent and -independent approach, with a particular focus on isolating and characterizing subdominant LAB species. In particular, three autochthonous LAB strains belonging to the species *Leuconostoc mesenteroides*, *Enterococcus durans*, and *Enterococcus faecalis* were characterized for their probiotic potential.

## 2. Materials and Methods

### 2.1. Sample Collection

NGN, New Generation Nutraceuticals Srl (Avellino, Italy) provided each type of waste—buffalo mozzarella cheese whey (CW) and ricotta cheese exhausted whey (RCEW). Three 1 L batches were collected from three separate production cycles on the same day at a dairy facility in the Campania region (Italy), using sterile disposable plasticware (Artiglass, Padua, Italy). Samples were refrigerated at 4 °C and transported to the laboratory within a few hours. Under sterile conditions, the three batches for each wastewater type were pooled (volume ratio 1:1:1) to obtain composite representative samples. Three aliquots of each pooled sample were taken and immediately employed for culture-dependent microbial analyses (three technical replicates). Three 100 mL aliquots of both CW and RCEW were frozen at −80 °C for the culture-independent analyses.

The proximal composition of the wastewaters collected was as follows: CW; proteins, 1.1% (w/vol); lactose, 4.0% (w/vol); fat, 1.2% (w/vol); ash, 0.5% (w/vol); RCEW; proteins, 0.3% (w/vol); lactose, 2.5% (w/vol); fat, 0.2% (w/vol); and ash, 0.5% (w/vol). The pH of CW and RCEW was 4.7 ± 0.1 and 4.3 ± 0.2, respectively.

### 2.2. Metabarcoding Analysis

#### 2.2.1. DNA Extraction

Frozen CW and RCEW were freeze-dried and DNA was extracted from the samples via the 3C-TAB procedure [14], based on the use of a 3C-TAB buffer [C-Tab I (C-Tab 4%); C-Tab II (C-Tab 10%, NaCl 0.7 M); and C-Tab III (C-Tab 1%, Tris–HCl 50 mM)] and solution 2A (NaCl 2.8 M, Tris–HCL 200 mM at pH 8, and EDTA 40 mM at pH 8). In detail, DNA extracted from the 150 mg and 106 mg samples obtained by freeze drying 2 mL of CW and RCEW, respectively, were placed in sterile 2 mL tubes and treated with RNase (20 mg/mL, Sigma-Aldrich, St. Louis, MO, USA). The quality/quantity was checked using 1% agarose gel electrophoresis separation and a spectrophotometric assay through NanoDrop (ThermoScientific, Waltham, MA, USA). DNA extraction and analysis were performed in two replicates.

#### 2.2.2. 16S and ITS rDNA PCR Amplification

PCR amplification was performed on 25 ng of DNA from each sample. For the analysis of the bacterial community, the universal primers 27-For. (5′-GAGATT TGATCCTGGCTCAG-3′) and 1495-Rev. (5′-CTACGGCTACC TTGTTACGA-3′), specific to the 16S rDNA region [15], were used. Primers ITS1 (5′-TCCGTAGGTGAACCTGCGG-3′) and ITS4 (5′-TCCTCCGCTTATTGATATGC-3′) were used to amplify the fungal ITS rDNA region [16]. Amplification was performed using Taq polymerase (Bioline, Meridian Bioscience, Cincinnati, OH, USA). The presence and purity of the PCR products were verified through gel electrophoresis in agarose gel (1%).

#### 2.2.3. Nanopore Sequencing

PCR amplicons of each sample were pooled using a ratio of 1:1 (amplicon 16S–amplicon ITS) and purified using AMPure beads. About 45 ng were used for library preparation using the Rapid Barcoding Kit 96 (SQK-RBK 110.96) and following the manufacturer instructions (Oxford Nanopore Technologies, Oxford, UK). The run was performed using a Flongle Flow Cell (R9.4.1) on a Mk1B device (Oxford Nanopore Technologies, UK). One overnight amplicon sequencing run was performed. Base calling and demultiplex were performed using MinKNOW (v22.08.9) and Guppy software (v6.2.11 + e17754e) [17], with default quality score thresholds and adapter trimming parameters. Only high-quality reads were retained for downstream analysis.

### 2.3. Microbiological Analyses

For the microbiological analyses, we followed the protocol of [18] with some modifications. Presumptive lactic acid bacteria (LAB) were determined on the Man–Rogosa–Sharpe (MRS) agar medium (Oxoid, Basingstoke, Hampshire, UK), a selective medium commonly used to promote LAB growth. To inhibit eukaryotic microorganisms, the medium was supplemented with cycloheximide (0.1 g/L, *w*/*v*), and the plates were incubated at 28 °C and 37 °C for 24 h in a gas-tight anaerobic jar system (Oxoid AnaeroJar, Thermo Fisher Scientific, UK) with anaerobic gas-generating sachets (Oxoid Anaero-Gen™, Thermo Fisher Scientific) to create an oxygen-free environment. This approach also allowed the recovery of subdominant lactic acid bacteria, including strains not detected through sequencing, possibly due to their low abundance. For yeast enumeration, yeast extract peptone dextrose (YPD) agar (Oxoid), a nutrient-rich medium suitable for a wide range of yeasts, supplemented with chloramphenicol (0.1 g/L, *w*/*v*) to suppress bacterial growth, was used. Incubation was carried out at 28 °C for 48 h. Cell density was determined by plating serial dilutions in three technical replicates.

### 2.4. Isolation and Identification of Lactic Acid Bacteria

For both media, colonies of bacteria and yeasts with different morphologies (e.g., size, shape, opacity) were picked and isolated from all dilutions [19]. In particular, thirty colonies of presumptive LAB were randomly selected from all the plates obtained for CW and RCEW dilutions, both at 28 and 37 °C. Gram-positive, catalase-negative, and non-motile rod and coccus isolates were cultivated in the corresponding broth media at 28 or 37 °C for 24 h and re-streaked onto the same agar medium. All isolates were preliminarily evaluated for the ability to acidify the MRS broth when incubated at 28 or 37 °C for 24 h. Only those able to acidify the culture medium were considered for further genomic DNA extraction.

DNA extraction of bacteria isolates was carried out using a microLYSIS Buffer (Labogen, Rho, Italy) following the manufacturer instructions, and 3 µL of genomic DNA suspension was used for the amplification of 16S ribosomal DNA (rDNA) in a Mastermix containing: 5 µL of 10× AccuBuffer (Meridian, Bioscience Inc., Cincinnati, OH, USA), 2 µL of 50 mM MgCl2, 0.5 µL of 100 mM dNTP MIX, 1 µL of each primer, and 1 µL of Accuzyme DNA Polymerase 2.5 U/µL. The universal bacterial primers P0 (5′-GAGAGTTTGATCCTGGCT-3′) and P6 (5′CTACGGCTACCTTGTTAC-3′), were used. The PCR protocol was as follows: 95 °C for 3 min + (95 °C for 15 s + 55 °C for 15 s + 72 °C for 2 min) × 30 + 72 °C for 10 min. The 16S rDNA amplification was verified by an agarose gel electrophoresis separation of the amplicons. The band of about 1500 bp was then purified using the Gel/PCR DNA fragments Extraction kit cat. DF 100 (Geneaid Biotech Ltd., New Taipei City, Taiwan) and sequenced in external service (Bio-fab Research, Rome, Italy). rRNA sequence alignments were carried out using the multiple-sequence alignment method, and identification queries were fulfilled by a BLAST search [20] in https://blast.ncbi.nlm.nih.gov/Blast.cgi?PROGRAM=blastn&PAGE_TYPE=BlastSearch&LINK_LOC=blasthome, accession date: 15 March 2024.

### 2.5. Phylogenetic Analysis

Phylogenetic relationships among the isolated and identified strains and other bacterial species were analyzed using 16S rRNA gene sequences. Sequence alignment, tree construction, and visualization were performed with the MEGA software, version 11 (Molecular Evolutionary Genetics Analysis, https://www.megasoftware.net) [21]. Sequence alignments were performed using ClustalW, which was also used to generate phylogenetic trees using the bootstrap method, with 1000 replications through the neighbor-joining statistical method.

### 2.6. Potential Probiotic Features

Three of the identified strains belonging to subdominant species (according to the nanopore metabarcoding results) were tested for their potential probiotic activity.

#### 2.6.1. Resistance to Lysozyme

Resistance to lysozyme was evaluated through a modified version of the protocol proposed by Samedi and Charles [22]. In detail, overnight cultures of the strains were centrifuged at 4000× *g* for 10 min at 4 °C. The resulting cellular pellets were washed two times with sterile phosphate-buffered saline (PBS, pH 7.2) and resuspended in 2 mL of Ringer’s solution (composition: 8.5 g/L of NaCl, 0.4 g/L of KCl, and 0.34 g/L of hydrated CaCl_2_·2H_2_O Sigma-Aldrich), pH 7.0. Two hundred microliters of the cell suspensions were inoculated (10% vol/vol) in sterile electrolyte solution (SES, 0.22 g/L of CaCl_2_, 6.2 g/L of NaCl, 2.2 g/L of KCl, 1.2 g/L of NaHCO_3_) in the presence of 100 or 50 mg/L of lysozyme (Sigma-Aldrich). Controls were obtained by inoculating microbial cells into SES without lysozyme. Viable cell counts (CFU/mL) were determined after 30 and 180 min of incubation at 37 °C by serial dilution and plating on MRS agar, followed by incubation at 37 °C for 48 h. Each experiment was carried out in biological triplicate, and results were expressed as percentage viability relative to initial CFU counts at time zero (t0).

#### 2.6.2. Tolerance to Acidic pH and Bile Salts

Resistance to low pH was evaluated in vitro, simulating stomach conditions in 3 h, the estimated time for digestion, where the environmental pH reaches a value of around 1.5/2.0 [23,24]. An overnight culture of each tested strain in MRS broth was first grown; then, cells were harvested by centrifugation (10 min, 2500× *g*) and washed in phosphate saline buffer (PBS). Cell suspensions in PBS (0.5 mL) were further diluted with PBS adjusted to pH 2 and 3 with HCl (final volume of 5 mL). The suspensions were incubated at 37 °C under stirring conditions. Viability (%) was determined by a plate count on MRS agar after 1, 2, and 3 h of incubation and expressed as follows:

$$\%\;\mathrm{viability}\;=\;\frac{\log\left(CFUNt\right)}{\log\left(CFUNi\right)}\times 100$$

where CFU_Ni_ and CFU_Nt_ represent the log CFU/mL at time 0 and after different time intervals, respectively. For resistance to bile salts, the protocol previously proposed by [25] and PBS added with 0.3 and 0.5% (w/vol) bile salts (Sigma-Aldrich) were used. Results (% viability) were expressed as described above.

#### 2.6.3. Antibiotic Resistance

The susceptibility to 15 antibiotics (Kariosafe SrL Loc. Sistiana, 41/D 34011 Duino Aurisina (TS) Italy) belonging to different groups according to their mechanisms of activity (inhibitors of cell wall synthesis, inhibitors of protein synthesis, and inhibitors of nucleic acid synthesis) (Table 1) was investigated. Strains were grown in MRS broth at 28 °C overnight and 200 µL of cellular suspension dilution (10^6^–10^7^ cells/mL) was used to inoculate the MRS agar plate. After inoculation, the plates were kept at room temperature for 1 h and sterile paper disks were dispensed on the surface of the solid media. Incubation was carried out at 28 °C for 24 h in aerobic conditions. Inhibition zones were then measured, and susceptibility was expressed in terms of resistance (R), moderate susceptibility (MS), and susceptibility (S) based on interpretative zone diameters (mm), as previously reported by Charteris et al. in 1998 [25,26].

#### 2.6.4. Antimicrobial Activity

Evaluation of antimicrobial activity was carried out using indicator strains *Pseudomonas aeruginosa* PA01, *Escherichia coli* NCIMB11943, *Bacillus cereus* NCIMB9373, and *Staphylococcus aureus* NCDO949 belonging to the culture collection of the Department of Biology and Biotechnology “C. Darwin” (Sapienza University of Rome, Italy) and routinely propagated in Luria–Bertani (LB) broth at 37 °C. Two hundred microliters of an overnight culture of the indicator strains were plated onto the surface of MRS plates and kept at room temperature for 1 h. Ten microliters of the LAB strain cultures were spotted on the plate. Also, the supernatants of the LAB 24 h and 48 h cultures, sterilized through filtration (Millipore, 0.22 µm pore size), were tested. The diameters (mm) of the inhibition zones were measured after 48 h of incubation at 37 °C.

#### 2.6.5. Early Adhesion and Biofilm Formation

The adhesion capacity of the LAB strains was assessed using the Clinical BioFilm Ring (cBRT) protocol, following the procedure described by [27]. *Lacticaseibacillus paracasei* I 1688, a strain with minimal adhesion capacity at 5 h, was employed as a negative control [28], while *Staphylococcus aureus* ATCC 6538, a well-established biofilm producer at 5 h, was used as positive control.

### 2.7. Statistical Analysis

All the analysis was carried out in triplicate. The data were subjected to statistical analysis, performed using Tukey’s multiple comparisons test. Significant differences are indicated by asterisks (* *p* < 0.05, ** *p* < 0.01, *** *p* < 0.001, **** *p* < 0.0001).

## 3. Results

### 3.1. Profiling of the Microbial Communities of Buffalo Mozzarella CW and RCEW

**Figure 1:**
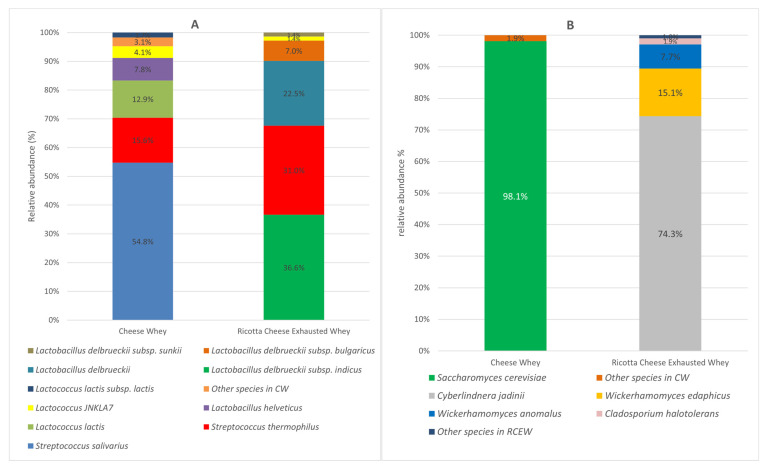
Microbial community composition of cheese whey and ricotta cheese exhausted whey. Bacteria species-level (**A**) and yeast and fungi species-level (**B**) expressed as relative abundance (%); species with a relative abundance of <1% are collected in “Other species”.

The microbial communities of both CW and RCEW included bacteria and eukaryotic microorganisms, primarily yeasts. The bacterial population of CW was predominantly characterized by the *Streptococcaceae* family, accounting for 86% of bacteria total abundance. Within this family, *Streptococcus* was the dominant genus (72%), followed by *Lactococcus*. *Streptococcus salivarius* (54.8%), *Streptococcus thermophilus* (15.6%), and *Lactococcus lactis* (12.9%) were identified as the primary species. The bacterial profile of RCEW exhibited a different distribution, with 68% of bacteria belonging to the *Lactobacillaceae* family and 30% to the *Streptococcaceae* family. Within the *Lactobacillaceae* family, *Lactobacillus delbrueckii* sp. was prominent, with *Lactobacillus delbrueckii subsp. indicus* accounting for 36.6% of the population. Notably, *Streptococcus thermophilus* was the sole representative of the *Streptococcaceae* family, constituting 31% of the bacterial community in RCEW. Among the species identified, *Streptococcus thermophilus* was the only one represented in both sample types. Moreover, the *Lactobacillus* species detected in CW were not found in RCEW (Figure 1A).

Regarding eukaryotes, the community was dominated by yeasts. *Saccharomyces cerevisiae* was the predominant species in CW, accounting for 98.14% of the total abundance. The remaining 1.86% comprised other yeast species (such as *Kluyveromyces marxianus*. Analysis of the RCEW revealed that *Cyberlindnera jadinii* was the most abundant species (74.3%), followed by *Wickerhamomyces edaphicus* (15.1%), *Wickerhamomyces anomalus* (7.7%), and *Cladosporium halotolerant* (1.9%) (Figure 1B).

### 3.2. Subdominant LAB Identification

The cell density of presumptive LAB in CW was 5.37 ± 0.12 and 5.11 ± 0.1 log CFU/mL, as, respectively, found in MRS after incubation at 28 and 37 °C, while significantly (*p* < 0.05) higher values were found in RCEW (8.15 ± 0.03 and 8.05 ± 0.15 log CFU/mL, respectively, at 28 and 37 °C). Also, yeasts were found at significantly lower densities in CW (6.14 ± 0.06 to 6.36 ± 0.02) compared with RCEW (8.05 ± 0.19), with no significant differences (*p* > 0.05) found among the data obtained at 28 or 37 °C for both the microbial groups.

**Figure 2:**
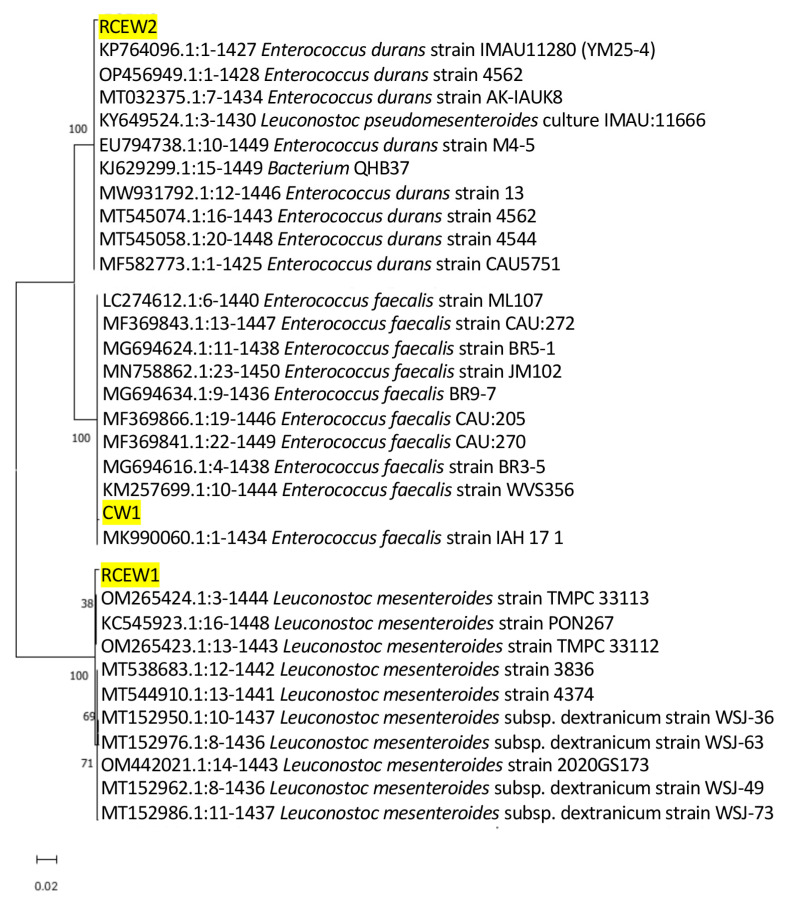
The phylogenetic tree of the LAB strains identified from buffalo cheese whey (CW1, RCEW1, and RCEW2, highlighted in the figure) and the ten most similar sequences obtained from BLAST analysis for each isolate is presented. The 16S rRNA sequences were aligned, and the phylogenetic relationships among them were constructed utilizing neighbor-joining analysis with MEGA version 11.0. The numbers at the nodes represent bootstrap values obtained from 1000 replications. GenBank accession numbers for 16S rRNA gene sequences are indicated. The scale bar of 0.02 denotes 2% nucleotide substitution.

Isolates obtained from the plate count of MRS were identified according to the 16S rDNA amplification and sequencing. The 15 isolates from CW were identified as *Streptococcus salivarius* (n.7), *Str. thermophilus* (n.4), and *Lactobacillus delbrueckii* subsp. *bulgarius* (n.3), while n.6 and n.7 isolates were identified, respectively, as *Lactobacillus delbrueckii* sp. and *Streptococcus thermophilus* in RCEW. These species were also detected by nanopore metabarcoding sequencing with a high abundance, suggesting that these species were the dominant ones. Three isolates belonged to species not revealed by metabarcoding sequencing: one isolate from CW, identified as *Enterococcus faecalis* (designated strain CW1), and two from RCEW, identified as *Leuconostoc mesenteroides* (designated strain RCEW1) and *Enterococcus durans* (strain RCEW2). It should be noted that all species with a relative abundance below 1% are grouped within the “others” category in the metabarcoding analysis report. Given the growing interest in exploring subdominant microbial species as potential sources of novel probiotic strains, these three isolates were selected for further characterization of their potential probiotic properties. Figure 2 illustrates the phylogenetic analysis based on the 16S rRNA genes of the aforementioned bacteria to elucidate the relationships between whey isolates and other bacterial strains already present in GenBank.

### 3.3. Potential Probiotic Features

The potential probiotic properties of *E. faecalis* CW1, *Ln. mesenteroides* RCEW1, and *E. durans* RCEW) were evaluated in vitro. First, their resistance to extreme conditions in the gastrointestinal tract (low pH in the stomach and bile salts in the upper intestine) and to lysozyme activity was assessed. Moreover, their antibiotic resistance and adhesion capacity were investigated.

#### 3.3.1. Resistance to Lysozyme

**Figure 3:**
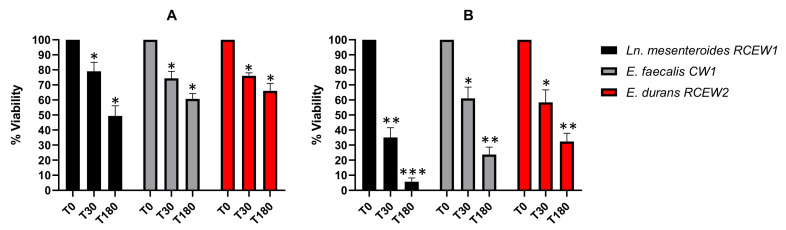
In vitro tolerance to lysozyme. (**A**) Bacteria viability is expressed as % relative to t0 after 30, and 180 min of exposure to 50 mg/L lysozyme in SES buffer. (**B**) Bacteria viability is expressed as % relative to t0 after 30 and 180 min of exposure in 100 mg/L of lysozyme in SES buffer. Statistical analysis was performed using Tukey’s multiple comparisons test. Significant differences are indicated by asterisks (* *p* < 0.05, ** *p* < 0.01, *** *p* < 0.001).

The high concentration of lysozyme in the oral cavity represents the initial barrier for probiotics immediately after administration. In Figure 3A, the LAB viability after 30 and 180 min of treatment with 50 mg/L lysozyme is reported. All the strains tested exhibited a substantial resistance to lysozyme after 30 min of incubation (78%, 73%, and 73%, respectively), with survival percentages decreasing after 180 min. In particular, *Ln. mesenteroides* RCEW1 was characterized by a lower resistance (48%) compared with *E. faecalis* CW1 (59%) and *E. durans* RCEW2 (66%) after 3 h of exposure. When the cells were subjected to a 100 mg/L lysozyme treatment (Figure 3B), the survival of *Ln. mesenteroides* RCEW1 was always lower than 10%, while *E. faecalis* CW1 maintained a viability of 66 and 59%, respectively, after 30 and 180 min. *E. durans* RCEW2 viability after 30 and 180 min of treatment was 68 and 51%, respectively.

#### 3.3.2. Tolerance to Acidic pH and Bile Salts

**Figure 4:**
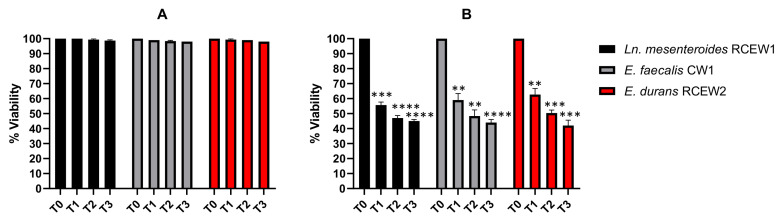
In vitro tolerance to gastric pH. Viable bacteria after 3 h of incubation in PBS adjusted to pH 3 (**A**) and pH 2 (**B**). Statistical analysis was performed using Tukey’s multiple comparisons test. Significant differences are indicated by asterisks (** *p* < 0.01, *** *p* < 0.001, **** *p* < 0.0001).

Exposure to pH 3 for 3 h did not affect the survival of any strain (Figure 4A), whereas at pH 2 (Figure 4B), the viability of *Ln. mesenteroides* RCEW1 decreased to 55% after 1 h and to 45% after 3 h of incubation. *E. faecalis* CW1 exhibited a viability of 59% after 1 h and 44% after 3 h of exposure. *E. durans* RCEW2 demonstrated a similar response, with a viability of 62 and 42%, respectively, after 1 h and 3 h of incubation. Treatment with bile salts at different concentrations was evaluated using the same method. At 0.3% concentration, the number of viable cells of all the tested strains remained higher than 6.50 Log CFU/mL until the end of the incubation (Table 2). However, when incubated in the presence of 0.5% bile salts, the number of viable cells decreased significantly for *Ln. mesenteroides* RCEW1 and *E. faecalis* CW1 (4.34 ± 0.06 and 3.99 ± 0.17 Log CFU/mL after 3 h, respectively), while *E. durans* RCEW2 exhibited a higher resistance (Table 2).

#### 3.3.3. Antibiotic Resistance

*Ln. mesenteroides* RCEW1 exhibited resistance to several cell wall synthesis inhibitors, including cefuroxime, cephalothin, ampicillin, cefotaxime, aztreonam, and vancomycin, as shown in Table 3. In contrast, the strain showed moderate susceptibility to penicillin G. *Ln. mesenteroides* RCEW1 is susceptible to inhibitors of protein synthesis such as erythromycin, chloramphenicol, clindamycin, and tetracycline. Within this antibiotic group, resistance was observed only for amikacin, whereas the strain was moderately susceptible to gentamicin and streptomycin. Inhibitors of cell wall synthesis were not effective against *E. faecalis* CW1. Indeed, resistance to cefuroxime, cephalothin, cefotaxime, and aztreonam (beta-lactam antibiotics) were observed for this strain, whereas it showed susceptibility to vancomycin and, to a lesser extent, to ampicillin and penicillin G. Resistance to different inhibitors of protein synthesis (gentamicin, amikacin, and streptomycin) was also observed for *E. faecalis* CW1, while it showed susceptibility to chloramphenicol, tetracycline, clindamycin, and erythromycin. *E. durans* RCEW2 exhibited resistance to cefuroxime, cephalothin, cefotaxime, and vancomycin; it showed susceptibility to ampicillin and penicillin G, as well as moderate susceptibility to aztreonam. Inhibitors of protein synthesis antibiotics were highly effective, and resistance was observed only for amikacin. All tested strains demonstrated susceptibility to rifampicin (inhibitor of nucleic acid synthesis).

#### 3.3.4. Antimicrobial Activity

**Figure 5:**
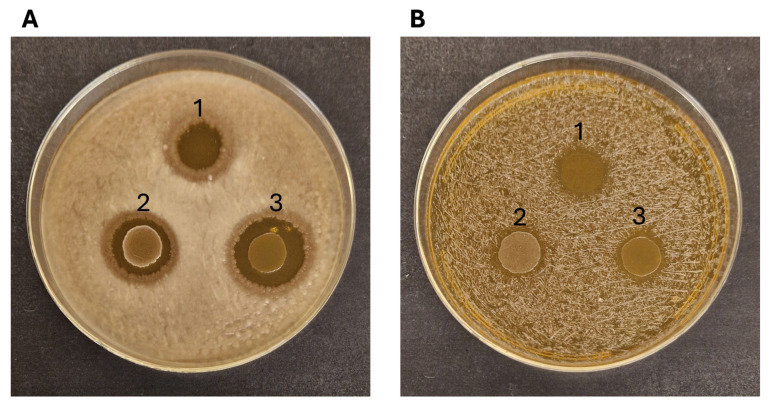
Antimicrobial activity of *Ln. mesenteroides* RCEW1 (1), *E. faecalis* CW1 (2) and *E. durans* RCEW2 (3) towards *B. cereus* NCIMB9373 (**A**) and *S. aureus* NCIMB949 (**B**).

All the LAB strains tested showed relevant antimicrobial activity towards the Gram-positive *B. cereus* NCIMB9373 and, to a lesser extent, against *Staphylococcus aureus* NCIMB949 (Figure 5). No effect was observed on *Pseudomonas aeruginosa* PA01 and *Escherichia coli* NCIMB11943. The supernatants of the bacterial cultures collected after 24 and 48 h showed no antibacterial activity.

#### 3.3.5. Early Adhesion and Biofilm Formation

**Figure 6:**
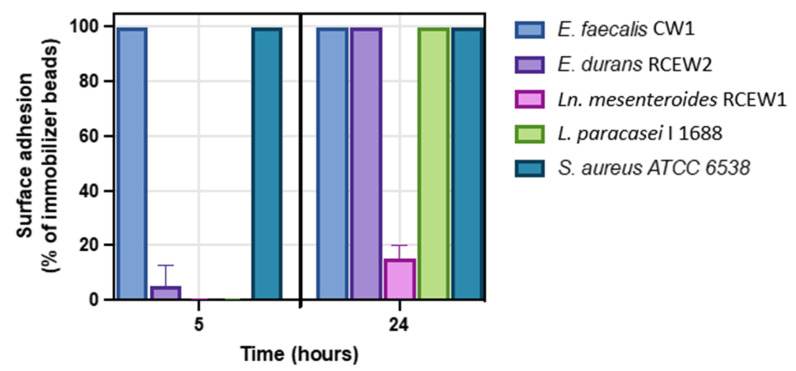
Biofilm formation at 5 h (early adhesion) and 24 h (mature biofilm) using the clinical biofilm ring test (cBRT) for *E. faecalis* CW1, *E. durans* RCEW2, *Ln. mesenteroides* RCEW1, *L. paracasei* I 1688, and *S. aureus* ATCC 6538. Error bars indicate the standard error of the mean from three independent experiments, carried out in duplicate.

The adhesion and biofilm-forming capabilities of the tested strains were evaluated at 5 and 24 h. Under the test conditions, after 5 h of incubation, no biofilm formation was observed for *Ln. mesenteroides* RCEW1. *E. durans* RCEW2 demonstrated limited biofilm production compared with *Lactobacillus paracasei* I 1688, which served as a negative control at this initial stage (Figure 6 and Figure S1). In contrast, *E. faecalis* CW1 exhibited significant biofilm formation, comparable to that of the positive control *Staphylococcus aureus* ATCC 6538. After 24 h, both *E. faecalis* CW1 and *E. durans* RCEW2 displayed robust biofilm formation, while *Ln. mesenteroides* RCEW1 was characterized by a low adhesion capacity throughout the assay.

## 4. Discussion

The search for potential probiotics among subdominant species in food microbiota is scientifically justified by their unique ecological roles and adaptive capabilities. While dominant species often shape the overall microbial environment, subdominant bacteria possess specialized functions that enhance microbial diversity, resilience, and host interactions [29]. Subdominant species may harbor unique metabolic traits, such as the production of antimicrobial and bioactive compounds, or enzymes that enhance gut health and digestion. Often, subdominant microorganisms evolved to persist in complex microbial communities, developing mechanisms for adhesion, stress resistance, and pathogen inhibition that are considered key traits for effective probiotics. Reduced risk of opportunistic pathogenicity is moreover associated with the subdominant component of microbiota, making them safer candidates for probiotic applications [30]. Exploring subdominant bacteria in fermented foods and other microbiomes expands the pool of potential probiotics beyond traditionally studied species, leading to innovative applications for human and animal health.

In this work, the microbiota of two dairy by-products derived from the Campania buffalo mozzarella cheese production chain were targeted as sources of biodiversity.

The uniqueness of the microbial communities detected in CW and RCEW is closely linked to the regional and artisanal characteristics of the Mozzarella di Bufala Campana PDO production process, including the mandatory use of a natural whey starter. This starter, obtained by the spontaneous fermentation of residual whey (*siero innesto*), introduces a highly diverse and site-specific microbiota into the production chain. Recent studies [18,31] have shown that this spontaneous fermentation leads to the enrichment of complex microbial consortia dominated by lactic acid bacteria, particularly *Streptococcus thermophilus*, *Lactobacillus delbrueckii*, and *Lactobacillus helveticus*, but also includes subdominant taxa such as *Leuconostoc* and *Enterococcus* spp. [5,31]. Overall, the technological and functional potential of the subdominant species in these microbial communities has only been partially investigated.

The composition of the microbial communities of the natural whey starter (and buffalo mozzarella) is strongly influenced not only by the raw buffalo milk microbiota, but also by environmental factors (e.g., air, water, and surfaces) and the repeated back-slopping practice used for whey fermentation, which promotes the maintenance of plant-specific microbial signatures. Levante et al. [5] demonstrated that the use of natural whey starters significantly affects microbial succession during curd fermentation and mozzarella formation, contributing to the development of distinct sensorial and functional profiles.

The microbial consortia developed during natural whey starter fermentation and mozzarella production evolve dynamically throughout the process and are ultimately reflected in by-products CW and RCEW. Obviously, the cell density of CW, which is heated to 80–90 °C to allow curd stretching, leads to a microbial population reduction, and the densities of both the bacteria and yeasts do not exceed 5.0 Log CFU/mL.

The metabarcoding analysis of CW showed a specialized bacterial community dominated by LAB. In particular, the dominance of the Streptococcaceae family (86% in total abundance) confirms the suitability of the environment and process conditions. *S. salivarius* [32] and *Streptococcus. thermophilus*, the most represented species, are commonly associated with fermented dairy products and have well-known technological significances [33]. As expected, the bacterial community in RCEW was markedly different from that of CW, as this by-product underwent additional intense thermal treatment and a progressive decrease in protein and lactose levels, resulting in the dominance of *Lactobacillaceae* (68% abundance). Nevertheless, the presence of *S. thermophilus* (31%) was also found in RCEW.

The community of yeasts also differed markedly between the two matrices. In CW, *Saccharomyces cerevisiae* was the dominant species, while the analysis of RCEW revealed a more complex composition, including several thermoresistant species: *Cyberlindnera jadinii* (the most abundant species), *Wickerhamomyces edaphicus* (15.1%), *W. anomalus* (7.7%), and *Candida halotolerans*, whose presence in fermented food matrices and resistance to intense processing were well documented [34,35,36].

The scientific literature largely discussed the limitations of nanopore metabarcoding in detecting low-abundance microorganisms within microbiota, due to different reasons, including: (i) a primer bias (different PCR primer efficiency, sequence read length, intrapopulation sequence and gene diversity) that can lead to the preferential amplification of certain taxa, causing an underrepresentation of those in low abundance); and (ii) bioinformatics pipelines that may struggle to differentiate between rare taxa and sequencing artifacts, leading to the potential misinterpretation of low-abundance reads [37,38,39,40].

To explore the potential of subdominant, low-abundance biotypes, only isolates belonging to species not detected by nanopore metabarcoding—due to their low cell counts and limited sequencing reads—were selected for probiotic characterization.

Several strains of *Ln. mesenteroides* [41], *E. durans* [42], and *E. faecalis* [43] have already been identified in the literature as potential probiotics. In this study, phylogenetic analysis revealed that *E. durans* RCEW2 was closely related to *E. durans* M4-5, a strain previously shown to alleviate intestinal inflammation [44]. To be defined as probiotics, strains must not be pathogenic; they must have a generally recognized as safe (GRAS) status and must be alive and viable in the human organism after intake, with at least 10^6^ to 10^9^ colony forming units (CFU) per day [45], so that they can colonize and exert the desired beneficial effects.

Initially, bacterial cells pass through the mouth, where saliva, containing lysozyme at concentrations of approximately 30–40 mg/L, represents the first barrier [46]. The *Enterococcus* strains CW1 and RCEW2 demonstrated a high resistance to 100 mg/L lysozyme. Evaluation of resistance to low pH and bile salts is essential for assessing the potential survival of these strains in the gastrointestinal environment. Passage through the stomach exposes the strains to gastric juices with a pH of 1.5–2 [23], followed by bile salts in the duodenum and small intestine. Low pH reduced viability by about 50% at pH 2 after 1 h. All strains showed a resistance to 0.3% bile salts after 3 h with a modest viability reduction, but only *E. durans* RCEW2 showed a significant resistance at the highest bile salt concentration tested as well.

Evaluating antibiotic resistance in putative probiotic bacteria is crucial for verifying potential horizontal gene transfers to human pathogens. Indeed, probiotic strains with intrinsic or non-transferable resistance can survive antibiotic treatments, maintaining gut microbiota balance during and after antibiotic use. This property may enhance their therapeutic effectiveness, especially in supporting recovery from infections or antibiotic-associated disturbances. However, it is crucial to ensure that resistance traits are not transferable to pathogenic bacteria, which could contribute to the spread of antibiotic resistance [41]. Among the LAB strains tested, *E. durans* RCEW2 showed the largest susceptibility, but some antibiotic resistance was observed in all strains. In particular, *Ln. mesenteroides* RCEW1, due to its cell wall structure, exhibited an intrinsic resistance to vancomycin, which was already reported as ineffective against this species [47,48]. Nevertheless, inhibitors of protein synthesis such as erythromycin, chloramphenicol, clindamycin, and tetracycline showed antimicrobial activity towards this strain and, in general, the species [49].

Key characteristics of probiotic microorganisms include adhesion capacity, which is crucial for host colonization, and the production of bacteriocin against human pathogens [44]. *E. faecalis* CW1 exhibited high biofilm production early, while both *E. faecalis* CW1 and *E. durans* RCEW2 demonstrated high surface adhesion after 24 h. On the other hand, *Ln. mesenteroides* RCEW1 was shown to be a weak biofilm producer even after 24 h, which implies a possible use of this strain that would guarantee rapid transit in the gastrointestinal tract, preventing it from colonizing the host.

The antimicrobial activity of the LAB strains was tested on indicators. Although the spectrum of the activity was complete for any of the strains tested, all demonstrated the capability to inhibit the growth of both the Gram-positive pathogenic indicators, *B. cereus* NCIMB9373 and *S. aureus* NCIMB949. The inhibition zones against *S. aureus* were smaller and less consistent than those observed against *B. cereus*, suggesting differential bacteriocin production or sensitivity [50,51], which should be further quantified in future work. Moreover, the fact that the supernatants were not active on the indicators, also suggests that the LAB may produce unstable antimicrobial compounds only when it is active and growing.

Table 4 summarizes the probiotic characteristics of the strains studied. Analyzing the overall results obtained from the in vitro evaluation of the probiotic properties of the tested strains, as reported in Table 4, it can be seen that the strains *E. faecalis* CW1 and *E. durans* RCEW2 possess the greatest numbers of these characteristics, including high adhesion ability. Low resistance to pH 2, common to all strains, could easily be bypassed by microencapsulation [52,53].

Historically, the most frequently utilized probiotic strains belong to the *Bifidobacterium*, *Lactobacillus*, and *Lactococcus* genera. However, the *Enterococcus* spp. are emerging as promising probiotic candidates and several studies indicated that some *Enterococcus* strains, following comprehensive characterization, may be suitable as safe probiotics in supplements [54,55,56]. Enterococci have many other important effects, such as modulating cheese microbiota and controlling pathogenic and spoilage microorganisms, as well as having beneficial effects on human host health [57]. They are also used in the treatment and prevention of certain human diseases, such as alleviating symptoms of irritable bowel syndrome, reducing antibiotic-induced diarrhea, and preventing various chronic intestinal diseases [58]. However, due to their ability to acquire genes through horizontal transfer, Enterococci can potentially develop various virulence determinants and become opportunistic pathogens. Due to these safety concerns, enterococci are not classified under generally recognized as safe (GRAS) in the United States [59,60]. This lack of a recognized safety status has hindered its use as an industrial food culture, despite its potential benefits [61,62].

## 5. Conclusions

This study provides insights into the microbial composition of cheese whey (CW) and ricotta cheese exhausted whey (RCEW), two dairy by-products derived from the traditional production process of Mozzarella di Bufala Campana PDO. Three subdominant strains, *E. faecalis* CW1, *E. durans* RCEW2, and *Ln. mesenteroides* RCEW1, exhibited in vitro probiotic features, including resistance to gastrointestinal stressors, antimicrobial activity, and adhesion or biofilm formation, particularly in *E. faecalis* CW1. From a practical perspective, these strains may be further developed for use in functional foods, nutraceutical formulations, or feed supplements aimed at supporting gut health and microbial balance. However, in the case of the *Enterococcus* spp., further genomic and functional safety evaluations are necessary to confirm the absence of transferable antibiotic resistance or virulence factors. Overall, the identification of functional microbial strains from CW and RCEW supports the sustainable reuse of dairy by-products and aligns with the principles of circular food systems and waste valorization in biotechnology.

## Figures and Tables

**Table 1:** Antibiotics and associated interpretative zone diameters for disk diffusion antibiotic susceptibility testing. Susceptibility was categorized as resistant (R), moderately susceptible (MS), or susceptible (S).

Antibiotic				Interpretative Zone Diameters (mm)		
Group	Name	Class/Subclass	Disk Concentration (µg)	R	MS	S
Inhibitors of cell wall synthesis	Cefuroxime	β-lactams/cephalosporins 2G	30	≤15	16–17	≥18
	Cephalothin	β-lactams/cephalosporins 1G	30	≤14	15–17	≥18
	Ampicillin	β-lactams/aminopenicillins	10	≤12	13–15	≥16
	Cefotaxime	β-lactams/cephalosporins 3G	30	≤14	15–22	≥23
	Aztreonam	β-lactams/monobactams	30	≤15	16–21	≥22
	Vancomycin	Glycopeptides	30	≤14	15–16	≥17
	Penicillin G	β-lactams/natural penicillins	10	≤19	20–27	≥28
Inhibitors of protein synthesis	Gentamicin	Aminoglycosides	10	≤12	-	≥13
	Chloramphenicol	Amphenicols	30	≤13	14–17	≥18
	Amikacin	Aminoglycosides	30	≤15	16–17	≥18
	Streptomycin	Aminoglycosides	25	≤11	12–14	≥15
	Tetracycline	Tetracyclines	30	≤14	15–18	≥19
	Clindamycin	Lincosamides	2	≤8	9–11	≥12
	Erythromycin	Macrolides	15	≤13	14–17	≥18
Inhibitors of nucleic acid synthesis	Rifampicin	Rifamycins	30	≤14	15–17	≥18

**Table 2:** Cell density (Log CFU/mL) of *Ln. mesenteroides* RCEW1, *E. faecalis* CW1, and *E. durans* RCEW2 after incubation in 0.3 and 0.5% bile salts in PBS (mean ± SD). Statistical analysis was performed using Tukey’s multiple comparisons test. Significant differences are indicated by asterisks (* *p* < 0.05, ** *p* < 0.01, *** *p* < 0.001, **** *p* < 0.0001).

Incubation Time (h)	*Ln. mesenteroides* RCEW1	*E. faecalis* CW1	*E. durans* RCEW2
0.3% bile salts in PBS			
0	7.14 ± 0.12	7.07 ± 0.10	7.11 ± 0.06
1	7.15 ± 0.05	6.98 ± 0.25	6.85 ± 0.22
2	6.81 ± 0.13 *	6.49 ± 0.20 *	6.85 ± 0.14
3	6.72 ± 0.12 *	6.50 ± 0.17 *	6.70 ± 0.20
0.5% bile salts in PBS			
0	6.57 ± 0.01	6.24 ± 0.05	7.06 ± 0.04
1	5.43 ± 0.1 **	4.88 ± 0.17 **	6.82 ± 0.06
2	4.49 ± 0.08 ***	4.46 ± 0.02 ***	6.08 ± 0.07 ***
3	4.34 ± 0.06 ****	3.99 ± 0.17 **	6.02 ± 0.06 ****

**Table 3:** Antibiotic resistances of *Ln. mesenteroides* RCEW1, *E. faecalis* CW1, and *E. durans* RCEW2. Results are expressed as R (resistant), MS (moderately susceptible), or S (susceptible).

Antibiotic	Interpretative Zone Diameters		
	*Ln. mesenteroides* RCEW1	*E. faecalis* CW1	*E. durans* RCEW2
*Inhibitors of cell wall synthesis*			
Cefuroxime	R	R	R
Cephalothin	R	R	R
Ampicillin	R	MS	S
Cefotaxime	R	R	R
Aztreonam	R	R	MS
Vancomycin	R	S	R
Penicillin G	MS	MS	S
*Inhibitors of protein synthesis*			
Gentamicin	MS	R	S
Chloramphenicol	S	S	S
Amikacin	R	R	R
Streptomycin	MS	R	S
Tetracycline	S	S	S
Clindamycin	S	S	S
Erythromycin	S	S	S
*Inhibitors of nucleic acid synthesis*			
Rifampicin	S	S	S

**Table 4:** Summary of the probiotic characteristics of the isolated strains indicating the presence (P) or absence (-) of the tested probiotic features.

		*Ln. mesenteroides* RCEW1	*E. faecalis* CW1	*E. durans* RCEW2
Lysozyme resistance	50	P	P	P
	100	-	P	P
Low pH resistance	3	P	P	P
	2	-	-	-
Bile salts resistance	0.3%	P	P	P
	0.5%	-	-	-
Antibiotic susceptibility	*In. cell wall synth.*	-	-	P
	*In. protein synth.*	P	P	P
	*In. nucleic acid synth.*	P	P	P
Antimicrobial activity	*B. cereus* NCIMB9373	P	P	P
	*S. aureus* NCIMB949	P	P	P
	*P. aeruginosa* PA01	-	-	-
	*E. coli* NCIMB11943	-	-	-
Adhesion ability	5 h	-	P	-
	24 h	-	P	P

## Data Availability

Sequencing dataset is available through the Sequence Read Archive (SRA) under accession PRJNA1220044. The 16S rRNA sequence of isolated bacteria are available through NBCI with the following accession numbers: PP494543.1; PP494544.1; PP494545.1.

## Supplementary Materials

The following supporting information can be downloaded at: https://www.mdpi.com/article/10.3390/microorganisms13081804/s1. Figure S1: Biofilm formation on polystyrene plates for *E. faecalis* CW1, *E. durans* RCEW2, *Ln. mesenteroides* RCEW1, *L. paracasei* I 1688, *S. aureus* ATCC 6538 after 5 h and 24 h of incubation at 37 °C. Images were obtained after magnetization of the plates on the Block Test and scanning with the Plate Reader. Negative controls with only BHI medium and magnetic microparticles are circled in red.

## Abbreviations

The following abbreviations are used in this manuscript:

CW	Cheese whey
RCEW	Ricotta cheese exhausted whey
