# Supplementary material for: Microbial Profiling of Buffalo Mozzarella Whey and Ricotta Exhausted Whey: Insights into Potential Probiotic Subdominant Strains
Source: Microorganisms. 2025 Aug 1;13(8):1804. doi: 10.3390/microorganisms13081804 (PMC12388355; doi:10.3390/microorganisms13081804)
Supplement: Supplementary file 1 [file microorganisms-13-01804-s001.zip › microorganisms-3733294-supplementary.pdf]

Supplementary materials

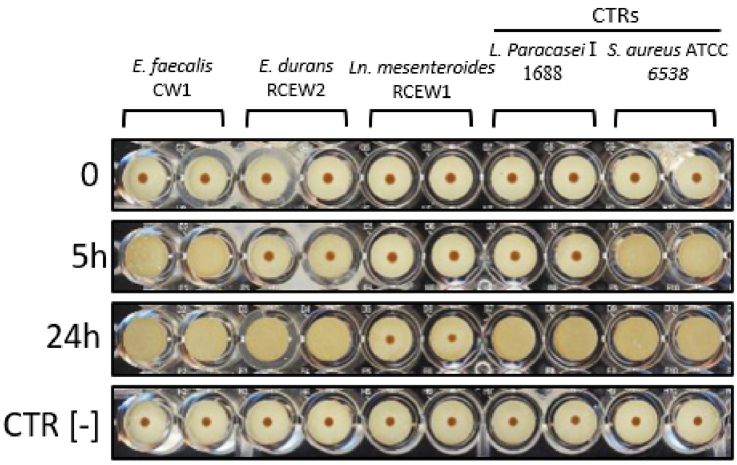

Figure S1 Biofilm formation on polystyrene plates for *E. faecalis* CW1, *E. durans* RCEW2, *Ln. mesenteroides* RCEW1, *L. paracasei* I 1688, *S. aureus* ATCC 6538 after 5 h and 24 h of incubation at 37°C. Images were obtained after magnetization of the plates on the Block Test and scanning with the Plate Reader. Negative controls with only BHI medium and magnetic microparticles are circled in red.
